# Comparison of New Technology Integrated and Nonintegrated Arterial Filters Used in Cardiopulmonary Bypass Surgery: A Randomized, Prospective, and Single Blind Study

**DOI:** 10.1155/2013/529087

**Published:** 2013-11-11

**Authors:** Özgür Gürsu, Selim Isbir, Koray Ak, Fethullah Gerin, Sinan Arsan

**Affiliations:** ^1^Department of Cardiovascular Surgery, Marmara University Medical Faculty, 34668 Istanbul, Turkey; ^2^Department of Medical Biochemistry, Marmara University Medical Faculty, 34668 Istanbul, Turkey

## Abstract

*Background*. Innovative cardiopulmonary bypass (CPB) settings have been developed in order to integrate the concepts of “surface-coating,” “blood-filtration,” and “miniaturization.” *Objectives*. To compare integrated and nonintegrated arterial line filters in terms of peri- and postoperative clinical variables, inflammatory response, and transfusion needs. *Material and Methods*. Thirty-six patients who underwent coronary bypass surgery were randomized into integrated (*Group In*) and nonintegrated arterial line filter (*Group NIn*) groups. Arterial blood samples for the assessments of complete hemogram, biochemical screening, interleukin-6, interleukin-2R, and C-reactive protein were analyzed before and after surgery. Need for postoperative dialysis, inotropic therapy and transfusion, in addition to extubation time, total amount of drainage (mL), length of intensive care unit, and hospital stay, and mortality rates was also recorded for each patient. *Results*. Prime volume was significantly higher and mean intraoperative hematocrit value was lower in *Group NIn*, but need for erythrocyte transfusion was significantly higher in *Group NIn*. C-reactive protein values did not differ significantly except for postoperative second day's results, which were found significantly lower in *Group In* than in *Group NIn*. *Conclusion*. Intraoperative hematocrit levels were higher and need for postoperative erythrocyte transfusion was decreased in Group In.

## 1. Introduction

Cardiopulmonary bypass (CPB) has a known association with various postoperative morbidities which have resulted in an increased need for research to be performed to find sources of formation and methods of reduction of the incidence of complications. It has been suggested that coated circuits improve postoperative outcomes compared with the traditional uncoated ones [1]. Different types of coating attenuate inflammatory response, platelet activation, fibrinolysis, need for transfusions, postoperative cerebral dysfunction, and myocardial damage compared with the “uncoated” technology [2]. Gaseous microemboli generated mainly by the cardiopulmonary bypass (CPB) circuit components have been promptly recognized as important contributing factors to embolic organ damage and, consequently, as specific triggers of systemic inflammatory response syndrome [3]. Accordingly, arterial line filters have been proposed to reduce the risk of embolism, and later on their antiembolitic efficacy has been confirmed. Finally, miniaturization of the CPB machine has proved to be effective in reducing pump-prime, inflammation, blood loss, and donor blood usage which are occasional outcomes of cardiopulmonary bypass surgery [4].

It is known that CPB induces a systemic inflammatory reaction that results in increased postoperative morbidity and hospital stay [5–7]. Activated leukocytes and platelets and their interaction play a pivotal role in inflammatory processes. The cells are activated by proinflammatory mediators such as complement, inflammatory cytokines, thrombin, reactive oxygen species, and endotoxin [5]. In the setting of CPB surgery, the artificial surface of the bypass and the membrane oxygenator are important sources for platelet and leukocyte activation [6]. It is well known that activated neutrophils infiltrate the myocardium and contribute to reperfusion injury, which occurs after reopening the aorta. CPB surgery has been shown to contribute to inflammatory cell activation [6, 7].

Innovative CPB settings have been developed in order to integrate the concepts of “surface-coating,” “blood-filtration,” and “miniaturization.” Indeed, the integration of arterial line filters with oxygenators fulfill these principles. However, there are a few studies that compare these innovations with traditional CPB equipment. The aim of the present study was to compare integrated and nonintegrated arterial line filters in patients who underwent CPB surgery in terms of peri- and postoperative clinical variables, inflammatory response, and transfusion needs.

## 2. Materials and Methods

### 2.1. Study Design

This study was approved by the local Institutional Review Board (09.2010.0095/B.30.2.MAR.0.01.02/AEK/55). Written informed consent was obtained from all subjects. Thirty-six consecutive patients, admitted to the Department of Cardiovascular Surgery of our university, between March 2011 and November 2011, were prospectively included in the study. Patients were divided into two groups by block randomization method using the sealed envelope technique. In the study, oxygenators with an integrated (*Group In*, *n* = 18; 50%) or nonintegrated (*Group NIn*, *n* = 18; 50%) arterial filters were used.

Patients who did not want to participate in the study, patients who required emergency surgery or additional surgical procedure due to any complications, the ones with known malignancies, hepatic and renal disorders, unstable angina, myocardial damage, and low cardiac reserve (left ventricular ejection fraction <40%), and the ones under 18 years old were excluded from the study. Patients were excluded from the study if they had systemic infections, urinary tract infections, concurrent use of antibiotics, or steroids as these conditions may affect serum levels of IL-6 and IL-2R.

### 2.2. Anaesthesia and Surgical Procedure

Anesthesia was induced with 4–6 mg/kg pentothal, 0.1 mg/kg midazolam, 3 *μ*g/kg fentanyl, and 0.1 mg/kg vecuronium bromide. After tracheal intubation, right atrium catheterization was performed for monitoring central venous pressure (CVP). Anesthesia was maintained with 10 *μ*g/kg fentanyl and 2 mg/kg propofol as infusion. 0.05 milligrams of vecuronium bromide and 0.08 mg/kg midazolam were given every 45 min throughout the operation.

All procedures were performed using an S5 roller pump (Stöckert, Sorin Group Inc., Munich, Germany) in both groups. Polyvinyl chloride tube was the same for both groups. In *Group NIn*, hollow-fibre membrane oxygenator (Dideco Compactflo, Sorin Biomedica, Mirandola, Italy) and arterial filter (Dideco, Sorin Biomedica, Mirandola, Italy) were used. The prime volume was 1650 mL of crystalloid solution containing 1000 mL Ringer's lactate, 500 mL gelofusine, and 150 mL mannitol. In *Group In*, oxygenator with an integrated arterial fitler (Sorin Synthesis, Sorin Group Spa, Milano, Italy) was used. The prime volume was 1450 mL of crystalloid solution containing 900 mL Ringer's lactate, 400 mL gelofusine, and 150 mL mannitole.

 All operations were performed through a standard median sternotomy incision. For the revascularization of the left anterior descending artery, left internal mammarian artery, and for other vessels, appropriately longer segments of vena saphena magna excised either from the right or left extremity were used. Pericardium was opened and elevated with slings, and then 300 U/kg heparin sodium was delivered. During CPB surgery, additional doses of heparin were administered in case of need to keep activated clotting time above 400 secs. Arterial flow was maintained with an aortic cannula (CalMed Lab, CA, USA) implanted inside ascending aorta, while for venous return two-way irrigation-aspiration cannula (CalMed Lab, CA, USA) positioned inside right atrium was used. A cannula was implanted in ascending aorta for cardioplegia and vent, and then the operation was proceeded with CPB. Moderate degrees of systemic hypothermia (28°C–32°C) were used intraoperatively. Following cross-clamping of ascending aorta, a blood cardioplegic solution (10 mEq MgSO_4_, 20 mEq potassium) warmed to the temperature of the pump was delivered at a dose of 10 mL/kg through an antegrade route to achieve cardiac arrest. When cross-clamping time exceeded 20 minutes, additional dose of cardioplegic solution was prepared anew and delivered through antegrade route. In both groups, pulsatile-flow cardiopulmonary bypass was performed. Extracorporeal circulation was started with nonpulsatile mode, at the time of aortic cross-clamping procedure was switched to pulsatile mode. When cross-clamp was removed, and left ventricular ejection started, nonpulsatile mode was resumed again. Distal anastomoses were performed using 7/0-8/0 prolene sutures dependent on the vascular structure, and then cross-clamp was removed. Heart rate was resumed to normal sinus rhythm spontaneously or via defibrillation. Proximal anastomoses were approximated with 6/0 prolene sutures, while the ascending aorta was partially clamped. CPB was terminated when esophageal temperature reached 37°C, and optimal cardiac conditions were achieved. Heparin was neutralized with protamine at a ratio of 1 : 1. After hemostatic control, drains were inserted into mediastinum and thoracal cavity. For the closure of the sternum, steel wire, and for subcutaneous layer, skin vicryl sutures were used. 

### 2.3. Outcome Parameters

Arterial blood samples for the assessments of complete hemogram (white blood cell (WBC) counts, hemoglobin (Hb), hematocrit (Htc), and platelet counts), creatinine, blood urea nitrogen (BUN), alanine aminotransferase (ALT), and aspartate aminotransferase (AST) were collected (1) before the operation, (2) at 24 hours, and (3) 2 and 4 days after the operation.

Serum samples for the analysis of interleukin-6 (IL-6) and interleukin-2R (IL-2R) were collected (1) preoperatively, (2) during immediate postoperative period and before heparin administration, and (3) at 6 and 24 hours after the operation. The serum samples were centrifuged at 5,000 g for 5 min and stored at −80°C until they were used. IL-6 and IL-2R levels were measured using the immulite automated chemiluminometer system (Immulite 2000 Analyzer, Siemens Healthcare Diagnostics Products Ltd. Llanberis, Gwynedd UK).

Arterial blood samples for the measurement of C-reactive protein (CRP) were collected (1) before the operation, (2) after the operation and before heparin administration, (3) at 6 and 24 hours, and (4) 2 and 4 days after the operation. CRP was measured by the CRP immunoturbidimetric method (CRPLX from Roche Diagnostics, Indianapolis, IN) on a COBAS INTEGRA 800 analyzer (Roche Diagnostics).

Need for postoperative dialysis, inotropic therapy and transfusion, in addition to extubation time, total amount of drainage (mL), length of intensive care unit, and hospital stay, and mortality rates were also recorded for each patient.

### 2.4. Statistical Analysis

Data were analyzed using the Statistical Package for Social Sciences (SPSS) software (version 19.0 for Windows). All differences associated with a chance probability of 0.05 or less were considered statistically significant. Continuous variables are presented as mean ± SD. A normal distribution of the quantitative data was checked using Kolmogorov-Smirnov test. Parametric tests were applied to data of normal distribution and nonparametric tests were applied to data of questionably normal distribution. Independent-samples *t*-test and Mann-Whiney *U*-test were used to compare independent groups. The distribution of categorical variables in both groups was compared using Pearson chi-square and Fisher's exact tests. Repeated measures ANOVA was used for testing overall group differences. The calculation of the sample size was based on a power analysis. At a power of 80% using a significance level of *P* < 0.05, the sample size required was 18 subjects per study group.

## 3. Results

Of the 36 patients (26 males, 10 females) whose charts were reviewed, the average age was 63.78 ± 10.37 (range 44 to 84) years. Baseline characteristics of both groups were comparable and shown in Table 1. Perioperative variables of both groups were also comparable and shown in Table 2. Number of bypass grafts, cross-clamping, cardiopulmonary bypass times, and hypothermia values were not significantly different between groups. Prime volume was significantly lower, while mean intraoperative hematocrit value was significantly higher in *Group NIn* (*P* < 0.001 and *P* < 0.01, resp.). Total chest tube drainage, extubation time, hospital stay, and inotropic therapy were not significantly different between groups. Need for erythrocyte transfusion was higher in *Group NIn* compared to *Group In *(2.44 ± 1.10 vs 1.72 ± 0.67, resp., *P* < 0.05). There were no significant differences between the two groups in terms of transfusions of fresh frozen plasma and thrombocyte suspension (*P* > 0.05). Need for intensive care unit stay was lower in *Group NIn* compared to *Group In* (1.5 ± 0.6 vs 2.3 ± 0.9, *P* < 0.05). Need for postoperative dialysis and mortality was not recorded for any of the patients in the study group. 

**Figure 1:**
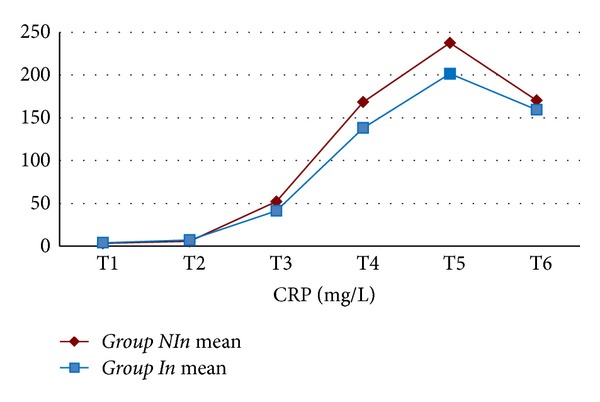
C-reactive protein levels of the study groups (T1: before the operation; T2: after the operation and before heparin administration; T3: 6 hours after the operation; T4: 24 hours after the operation; T5: 2 days after the operation; T6: 4 days after the operation).

**Figure 2:**
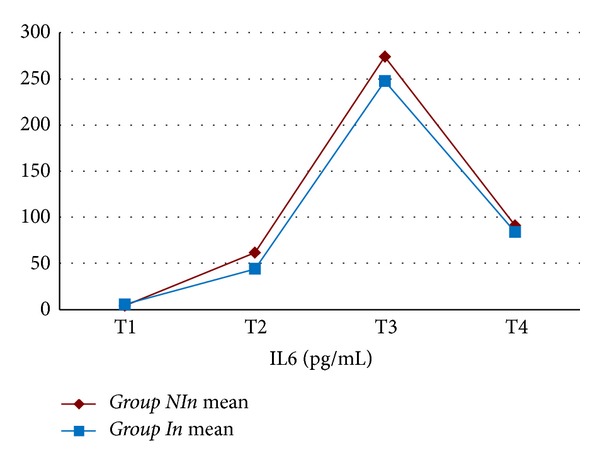
Interleukin-6 levels of the study groups (T1: before the operation; T2: after the operation and before heparin administration; T3: 6 hours after the operation; T4: 24 hours after the operation).

**Figure 3:**
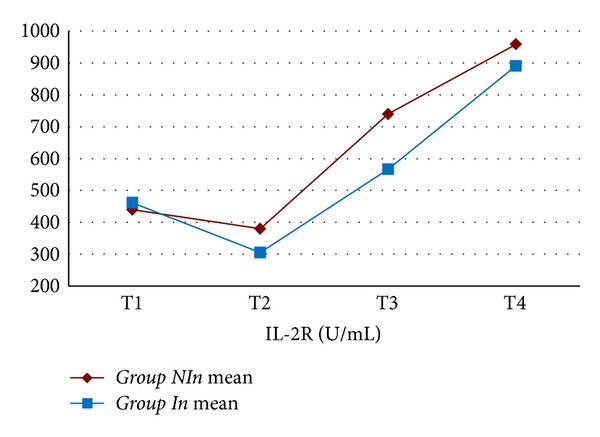
Interleukin-2R levels of the study groups (T1 = before the operation; T2 = after the operation and before heparin administration; T3 = 6 hours after the operation; T4 = 24 hours after the operation).

The mean value for WBC, Hb, Htc, platelet count, creatinine, BUN, ALT, and ASTwas not statistically different between the two groups. In both groups, CRP values increased statistically significantly starting from the 6th postoperative hour (*P* < 0.001) (Figure 1). When both groups were compared, CRP values were found to be comparable (excluding postoperative 2nd day); however on postoperative 2nd day, CRP values in *Group In *were statistically significantly lower when compared with *Group NIn *(*P* < 0.05) (Table 3). In both groups, IL-6 values started to increase following cardiopulmonary bypass, reached peak values at postoperative 6th hours, and then began to decline (Figure 2). Mean IL-6 values determined preoperatively, following cardiopulmonary bypass, and at postoperative 6th and 24th hours were not statistically significantly different between both groups (*P* > 0.05) (Table 4). In both groups, IL-2R values started to rise following cardiopulmonary bypass, and this increase continued up to postoperative 24th hour (Figure 3). IL-2R values determined preoperatively, following cardiopulmonary bypass, and at postoperative 6th and 24th hours were not statistically significantly different between both groups (*P* > 0.05) (Table 4).

**Figure 4:**
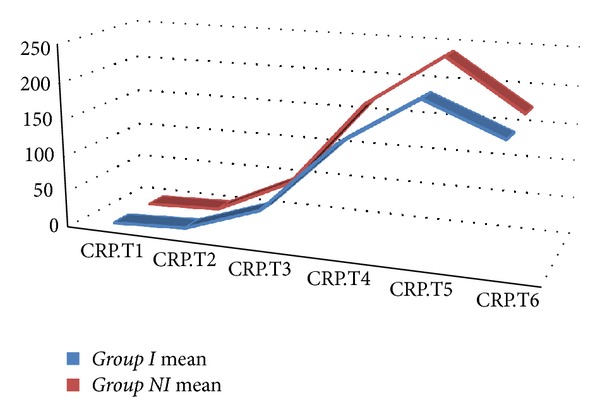
Area under the curve values of C-reactive protein.

**Figure 5:**
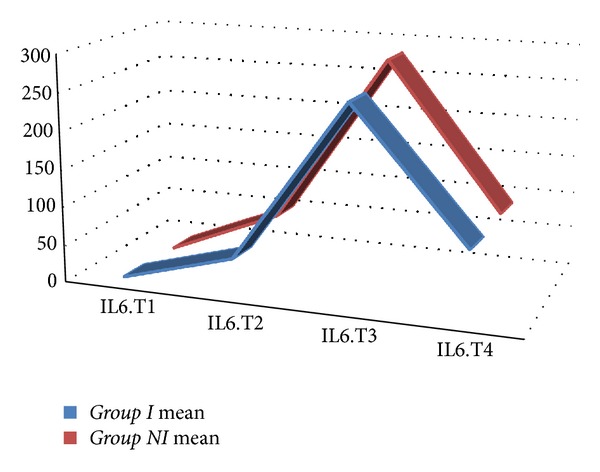
Area under the curve values of interleukin-6.

**Figure 6:**
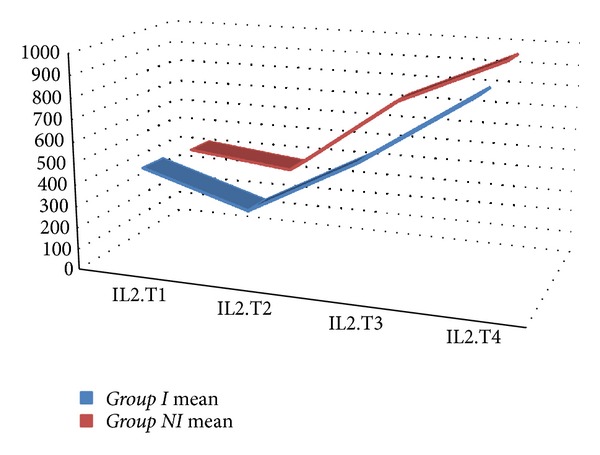
Area under the curve values of interleukin-2R.

Mean release of CRP, IL-6, and IL-2R over the entire time of observation by repeated measures ANOVA did not reveal any statistical significance. Mean CRP values were 159.44 ± 56.70 in *Group In* and 170.33 ± 54.01 in *Group NIn* (*P* = 0.211). Mean IL-6 values were 556.08 ± 68.19 in *Group In* and 629.47 ± 68.19 in *Group NIn* (*P* = 0.339). Mean IL-2R values were 95.34 ± 13.97 in *Group In* and 107.69 ± 13.97 in *Group NIn* (*P* = 0.596). Area under the curve values of CRP, IL-6, and IL-2R did not differ significantly between groups (Table 5) (Figures 4, 5, and 6). 

## 4. Discussion 

In our study, we evaluated the impact of integrated arterial filtration systems on systemic inflammatory response and postoperative requirement for transfusion. Employment of integrated filtration systems decreased prime volume for an amount of 200 cc which led to higher intraoperative hematocrit levels and lower postoperative requirement for the infusion of erythrocyte suspensions. In the group of patients in whom integrated arterial filters were used, detection of partially lower mean postoperative IL-6, IL-2R, and CRP values at all time points of blood collection promises potential gains in the future. 

During extracorporeal circulation, when blood comes in contact with nonendothelial surfaces, complement, and proinflammatory mediators such as cytokines are activated leading to emergence of a systemic inflammatory response. Systemic inflammatory response developing post-CPB can be restricted to an extent of only a subclinical increase in the levels of inflammatory mediators or it can be severe enough to cause multiorgan dysfunction or even death [8]. Many pharmacological and nonpharmacological preventive measures have been tried to attenuate post-CPB systemic inflammatory response. Coating of extracorporeal system components with biocompatible molecules, minimization of interfaces of extracorporeal circuits, and thus enabling to perform cardiac operations using lower prime volumes can be listed as some of the nonpharmacological preventive measures [9, 10]. Currently used conventional CPB systems have recently started to be integrated to oxygenators with arterial filters. This modification at a certain extent decreases the interface area between synthetic material and contact area leading to reduction in the amount of prime volume of the system [11]. 

During CPB, the total white blood cell count and the number of circulating neutrophils increase [12]. Morse and colleagues detected overall neutrophil and platelet activation but were unable to demonstrate an augmented activation of neutrophils and platelets drawn from the coronary microvasculature compared with a peripheral source. No correlation between duration of cross-clamping and cell activation was found [13]. Platelets deposited on endothelial cells recruit leukocytes at shear rates that otherwise do not allow leukocyte adhesion [14]. Rinder et al. reported that, in addition to an increased number of activated leukocytes, the number of leukocyte-platelet conjugates was increased in patients undergoing cardiac surgery with CPB [15]. In the present study, WBC, Hb, Htc, platelet count, creatinine, BUN, ALT, and AST were not statistically different between two groups.

Hemodilutional anemia occurring during CPB is an important cause of morbidity and mortality [16, 17]. To prevent related complications, circuits with smaller interface area which enable to work with lesser prime volumes have been tried to be designed. Decreasing prime volumes in cardiovascular systems diminishes intraoperative hemodilution and resultant need for transfusion [18, 19]. Remadi et al. compared minimal extracorporeal circuits with standard CPB circuits and found more than 2-fold difference in prime volumes between two groups which led to a rise in intraoperative hematocrit values and consequent decrease in the requirement for transfusion [20]. In a comparable study performed by Stalder et al., prevention of intraoperative excessive hemodilution led to a decrease in transfusion rates [21]. Ševerdija et al. used retrograde autologous priming method, obtained more than 2-fold gain in prime volumes, and demonstrated that use of this method increased intraoperative hematocrit values and also decreased requirement for transfusion [22]. In the present study, group in whom integrated arterial filters were used, a prime volume of 1450 cc was used, while in the nonintegrated group a prime volume of 1650 cc was used. Also in this study, the integrated arterial filter users had statistically significantly higher intraoperative hematocrit values and relatively lesser requirement for erythrocyte suspensions during the postoperative period when compared with those of nonusers.

In separate studies where Formes et al. evaluated post-CPB development of inflammatory response and Ak et al. investigated lipoprotein lipase polymorphism and its effects on atherosclerosis, the authors detected peak levels of IL-6 at post-CPB 6th hours [9, 23]. However, Delannoy et al. revealed that CPR reached its peak levels on post-CPB 2nd, and 3rd days [24]. Alataş et al. evaluated myocardial ischemia reperfusion damage developed after CPB and demonstrated an increasing trend in IL-2R levels even after postoperative 24th hours [25]. In the present study, we analyzed CRP, proinflammatory cytokine IL-6, and IL-2 receptors in order to assess the inflammatory response. In our study, we witnessed rapid rise in CRP, IL-6, and IL-2R values after CPB. IL-6 levels reached their peak levels at postoperative 6th hour with an increasing trend in successive measurements up to postoperative 24th hour. We also noted that CRP levels reached their peak values on postoperative 2nd day. Our results were in concordance with those of the literature. When we assess our study globally, even though postoperative IL-6, IL-2R, and CRP values were lower in the integrated arterial filter users, a significant difference between both groups could not be detected as for systemic inflammatory response. This phenomenon might be attributed only to biocompatibility of our oxygenator, normal-sized tubing we used which limited the biocompatible area. In other words, we think that combined use of biocompatible oxygenator and tubing sets and resultant increase in the biocompatible surface area of the circuit might accentuate anti-inflammatory effect. 

Our study has several limitations. Firstly, retrograde autologous prime volumes could be used to further decrease prime volumes which we already lowered using integrated arterial filters. Within this context, combination of integrated arterial filter systems with retrograde autologous prime method might be beneficial both in the alleviation of systemic inflammatory response and further decline in the requirement for transfusion. Secondly, the study was a single-centered investigation conducted with a small group of patients. Even though in a single-centered study all patients were operated by the same surgical and anesthesia team which provides concordance among interventions, and scarce number of patients appears to be the weak point of the study. Third limitation is the enrolment of a patient group with a relatively lower risk. Starting from the fact that open heart surgery can be performed in a patient with a lower risk using standard methods with decreased morbidity and mortality rates, it can be conceived that more valuable gains can be obtained in patients with a higher risk. 

Since lesser amounts of prime volumes were required for the patient in whom integrated arterial filter oxygenators were used, their intraoperative hematocrit levels were found to be relatively higher. Besides, the need for postoperative erythrocyte transfusion was also decreased. It has been detected that using integrated arterial filter has no significant effect on postoperative systemic inflammatory response.

## Figures and Tables

**Table 1:** Baseline characteristics of the study groups.

	*Group NI *(*n* = 18)	*Group I *(*n* = 18)	*P* value
Age (yr)	63.1 ± 11.3	64.4 ± 9.7	*P* > 0.05
Female/male	5/13	5/13	*P* > 0.05
Height (cm)	168.2 ± 6.3	167.7 ± 7.1	*P* > 0.05
Weight (kg)	82.1 ± 12.5	76.0 ± 11.9	*P* > 0.05
Diabetes mellitus	8/18	8/18	*P* > 0.05
Hypertension	13/18	14/18	*P* > 0.05
Hyperlipidemia	6/18	9/18	*P* > 0.05
Tobacco use	9/18	13/18	*P* > 0.05
Alcohol use	1/18	1/18	*P* > 0.05
CCS grading			
I-II	17/18	17/18	*P* > 0.05
III-IV	1/18	1/18	*P* > 0.05
NYHA classification			
I-II	15/18	17/18	*P* > 0.05
III-IV	3/18	1/18	*P* > 0.05
Preoperative ejection fraction (%)	61.3 ± 6.1	60.2 ± 5.5	*P* > 0.05

CCS: Canadian cardiovascular society; NYHA: New York heart association.

**Table 2:** Perioperative and postoperative variables of the study groups.

	*Group NI *(*n* = 18)	*Group I *(*n* = 18)	*P* value
Perioperative variables			
Number of bypass grafts	2.7 ± 1.1	3.3 ± 1.0	*P* > 0.05
Cross-clamp time (min)	46.6 ± 15.8	46.4 ± 14.1	*P* > 0.05
CPB time (min)	94.2 ± 30.9	93.6 ± 21.0	*P* > 0.05
Primary volume (mL)	1650	1450	*P* <0.001
Intraoperative Htc	22.1 ± 1.5	23.5 ± 1.1	*P* <0.01
Hypothermia (°C)	29.9 ± 1.8	30.7 ± 1.8	*P* > 0.05
Red cell transfusion (units)	2.44 ± 1.09	1.72 ± 0.67	*P* < 0.01
Postoperative variables			
Total drainage (mL)	597.2 ± 250.6	456.9 ± 175.7	*P* > 0.05
Extubation time (hours)	8.9 ± 1.9	11.7 ± 8.8	*P* > 0.05
Intensive care unit stay (days)	1.5 ± 0.6	2.3 ± 0.9	*P* <0.05
Hospital stay (days)	8.8 ± 4.3	9.5 ± 2.9	*P* > 0.05
Inotropic therapy	4/18	4/18	*P* > 0.05

Htc: hematocrit; CPB: cardiopulmonary bypass.

**Table 3:** C-reactive protein levels of the study groups.

	Time	*Group NI *(*n* = 18)	*Group I *(*n* = 18)	*P* value
C-reactive protein (mg/L)	T1	3.2 ± 1.5	4.0 ± 1.5	*P* > 0.05
	T2	5.6 ± 5.6	7.2 ± 7.6	*P* > 0.05
	T3	52.0 ± 50.3	41.3 ± 36.5	*P* > 0.05
	T4	168.4 ± 42.2	138.1 ± 51.8	*P* > 0.05
	T5	237.4 ± 38.7	201.7 ± 49.2	*P* < 0.05
	T6	170.3 ± 54.0	159.4 ± 56.7	*P* > 0.05

T1: before the operation; T2: after the operation and before heparin administration; T3: 6 hours after the operation; T4: 24 hours after the operation; T5: 2 days after the operation; T6: 4 days after the operation.

**Table 4:** Interleukin-6 and interleukin-2R levels of the study groups.

	Time	*Group NI *(*n* = 18)	*Group I *(*n* = 18)	*P* value
Interleukin-6 (pg/mL)	T1	4.0 ± 2.0	5.5 ± 2.7	*P* > 0.05
	T2	61.6 ± 50.1	44.1 ± 35.5	*P* > 0.05
	T3	274.1 ± 262.1	247.7 ± 171.5	*P* > 0.05
	T4	91.0 ± 40.5	84.1 ± 41.7	*P* > 0.05

Interleukin-2R (U/mL)	T1	439.5 ± 156.1	461.6 ± 197.2	*P* > 0.05
	T2	379.9 ± 286.6	305.4 ± 123.8	*P* > 0.05
	T3	739.8 ± 466.2	566.8 ± 343.0	*P* > 0.05
	T4	958.7 ± 453.2	890.6 ± 493.4	*P* > 0.05

T1: before the operation; T2: after the operation and before heparin administration; T3: 6 hours after the operation; T4: 24 hours after the operation.

**Table 5:** Area under the curve values of C-reactive protein, interleukin-6, and interleukin-2R.

	*Group NI *(*n* = 18)	*Group I *(*n* = 18)	*P* value
C-reactive protein	123.44 ± 113.33	102.44 ± 93.12	0.757
Interleukin-6	188.81 ± 167.43	166.71 ± 153.42	0.874
Interleukin-2R	626.12 ± 198.45	568.16 ± 148.35	0.706
